# Structural and DNA-binding properties of the cytoplasmic domain of *Vibrio cholerae* transcription factor ToxR

**DOI:** 10.1016/j.jbc.2021.101167

**Published:** 2021-09-04

**Authors:** Nina Gubensäk, Evelyne Schrank, Christoph Hartlmüller, Christoph Göbl, Fabio S. Falsone, Walter Becker, Gabriel E. Wagner, Sergio Pulido, N. Helge Meyer, Tea Pavkov-Keller, Tobias Madl, Joachim Reidl, Klaus Zangger

**Affiliations:** 1 Institute of Chemistry/Organic and Bioorganic Chemistry, University of Graz, Graz, Austria; 2 Institute of Molecular Biosciences, University of Graz, Graz, Austria; 3 Center for Integrated Protein Science Munich (CIPSM) at the Department of Chemistry, Technische Universität München, Garching, Germany; 4 Department of Pathology and Biomedical Science, University of Otago Christchurch, Christchurch, New Zealand; 5 Institute of Pharmaceutical Sciences/Pharmaceutical Technology and Biopharmacy, University of Graz, Graz, Austria; 6 Department of Medical Biochemistry and Biophysics, Karolinska Institute, Solna, Sweden; 7 Diagnostic and Research Institute of Hygiene, Microbiology and Environmental Medicine, Medical University of Graz, Graz, Austria; 8 Division of Experimental Allergology and Immunodermatology, University of Oldenburg, Oldenburg, Germany; 9 BioTechMed-Graz, Graz, Austria; 10 Field of Excellence BioHealth, University of Graz, Graz, Austria; 11 Gottfried Schatz Research Center for Cell Signaling, Metabolism and Aging, Institute of Molecular Biology & Biochemistry, Medical University of Graz, Graz, Austria

**Keywords:** *Vibrio cholerae*, nuclear magnetic resonance (NMR), protein interaction, transcription factor, membrane protein, CSP, chemical shift perturbation, CT, cholera toxin, TCP, toxin coregulated pilus, wHTH, winged helix–turn–helix

## Abstract

ToxR represents an essential transcription factor of *Vibrio cholerae*, which is involved in the regulation of multiple, mainly virulence associated genes. Its versatile functionality as activator, repressor or coactivator suggests a complex regulatory mechanism, whose clarification is essential for a better understanding of the virulence expression system of *V. cholerae*. Here, we provide structural information elucidating the organization and binding behavior of the cytoplasmic DNA-binding domain of ToxR (cToxR), containing a winged helix–turn–helix (wHTH) motif. Our analysis reveals unexpected structural features of this domain expanding our knowledge of a poorly defined subfamily of wHTH proteins. cToxR forms an extraordinary long α-loop and furthermore has an additional C-terminal beta strand, contacting the N-terminus and thus leading to a compact fold. The identification of the exact interactions between ToxR and DNA contributes to a deeper understanding of this regulatory process. Our findings not only show general binding of the soluble cytoplasmic domain of ToxR to DNA, but also indicate a higher affinity for the *toxT* motif. These results support the current theory of ToxR being a “DNA-catcher” to enable binding of the transcription factor TcpP and thus activation of virulence-associated *toxT* transcription. Although, TcpP and ToxR interaction is assumed to be crucial in the activation of the *toxT* genes, we could not detect an interaction event of their isolated cytoplasmic domains. We therefore conclude that other factors are needed to establish this protein–protein interaction, *e.g.*, membrane attachment, the presence of their full-length proteins and/or other intermediary proteins that may facilitate binding.

Cholera is a severe diarrheal disease caused by the Gram-negative bacterium *Vibrio cholerae*. The ability of the bacterium to survive even under harsh and low-nutrient conditions explains its persistence in aquatic environments between outbreaks of the disease, even outside of endemic regions (1, 2). Cholera outbreaks are still reported on a regular basis and the disease is epidemic in several regions of the world (3), emphasizing the importance of research in this field. Still numerous questions regarding the regulation of the virulence factor production in *V. cholerae* remain unanswered.

The main virulence factors in *V. cholerae* are the toxin coregulated pilus (TCP), which is required for the adherence of the bacterium in the small intestine (4) and the cholera toxin (CT), causing the main symptom of the disease, the fatal diarrhea (4, 5). The expression of the virulence factors is controlled by the ToxR regulon (6), involving numerous proteins, among them the inner membrane protein ToxR (7). ToxR plays a crucial role in *V. cholerae* since it controls the transcription of several genes in supposedly different ways, acting as an activator or a repressor, alone or in combination with a coactivator (8, 9, 10, 11, 12, 13).ToxR consists of a sensory periplasmic domain, a transmembrane region, and a cytoplasmic DNA-binding part (14, 15). The cytoplasmic domain of ToxR (cToxR) is homologous to OmpR, which is a member of the winged helix–turn–helix (wHTH) family of transcription factors and regulates the expression of the porin genes in *Escherichia coli* (16). ToxR regulates gene transcription *via* binding of its cytoplasmic domain to the so-called “tox-boxes” (17, 18, 19, 20).

Two of the genes directly controlled by ToxR are *ompU* and *ompT*, encoding two major outer membrane proteins (21, 22). As far as *ompU* is concerned, ToxR is an activator, reciprocal to *ompT* whose transcription is repressed upon binding of ToxR (23). ToxR is also involved in the activation of the *toxT* genes encoding a main transcription factor of *V. cholerae*, which directly controls the production of the main virulence factors CT and TCP (13, 24, 25, 26, 27). The current model proposes that the role of ToxR in the activation of the *toxT* genes is to enhance the activity of TcpP (28), another transmembrane “ToxR-like” receptor, which itself seems to be a relatively weak DNA binder (26, 29). During this activation process ToxR and TcpP interact directly (19, 30), probably *via* their cytoplasmic wing domains (19) and their periplasmic sensory domains (30). Furthermore, overexpression of ToxR leads to the activation of the transcription of the *ctxA* genes, encoding for the cholera toxin subunit A (31). Nevertheless, under physiological conditions, the *ctxA* promoter is controlled by ToxT only (8, 32, 33, 34, 35).

Following our recently published structural and functional investigation of the periplasmic domain of ToxR (36), we are aiming to shed light on the versatile functionality of its cytoplasmic DNA binding domain. To address this question we were interested in solving the structure of the cytoplasmic domain of ToxR (cToxR) as well as studying its binding behaviour to different DNA motifs of *V. cholerae* operons, which are activated, repressed, or coactivated by ToxR. The presented structure of cToxR confirms the presence of the predicted wHTH motif and furthermore reveals a topology different from other wHTH proteins, but similar to the “ToxR-like” protein CadC (37). Additionally, we could detect a general binding of the soluble form of cToxR to DNA, with the highest binding affinity for the *toxT* motif for whose activation cToxR acts as a “DNA-catcher” (19, 20, 26). The NMR guided HADDOCK (38, 39) model of cToxR bound to the *ompU* motif reveals further insights into the interaction between cToxR structural elements and the minor and major groove of the DNA. Finally, since we could not detect a direct interaction of the soluble cytoplasmic domains of TcpP and ToxR in NMR experiments, we conclude that the presence of the periplasmic and transmembrane domains could be essential for the protein–protein interaction, which is crucial for the activation of *toxT*.

## Results and discussion

### cToxR contains a wHTH motif including an α-loop

We first expressed, purified, and acquired NMR experiments on the complete cytoplasmic domain of ToxR (cToxR), consisting of 144 amino acids (Fig. S1). The 2D ^15^N-^1^H HSQC spectrum of cToxR revealed severe overlap in the unstructured region of the spectra, which complicated peak assignments. For this reason, we generated C-terminal truncated constructs of cToxR and recorded several 2D ^15^N-^1^H HSQC experiments. Finally, for the determination of the structure of the cytoplasmic DNA-binding domain of *V. cholerae* ToxR, we used a 134 amino acids long construct, referred to as cToxR_1–134, missing 49 C-terminal residues (Figs. S1 and S2 and Table S1).

Because of the high structural similarity between cToxR and wHTH protein cYycF (37) (PDB code: 2d1v), we decided to use a combination of NMR assignments (Table S1), including structural important NOEs (Table S2), chemical shift-based secondary structure predictions (40) (Fig. S3), and CS-Rosetta (41, 42). The calculation was performed for rigid residues I16-E128.

**Figure 1:**
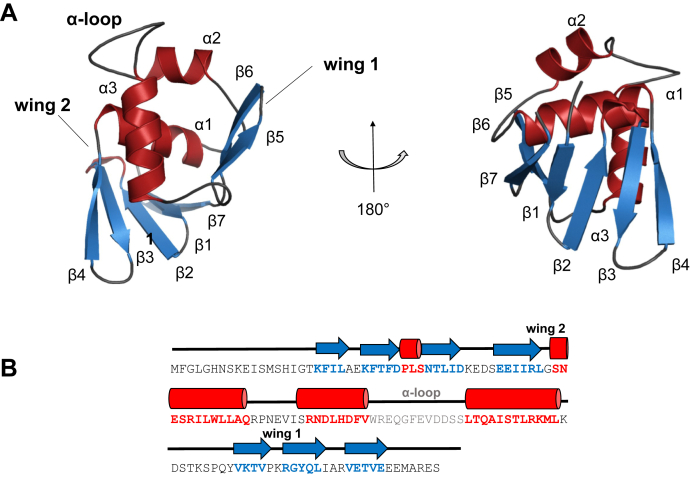
**cToxR_1-134 forms a winged helix-turn-helix (wHTH) motif*.****A*, two views of the NMR-Rosetta structure of cToxR_1–134. The structure is comprised of four N-terminal β strands (β1- β2- β3- β4) forming a five stranded β sheet with strand 7 (β7), followed by helix 1 (α1), a HTH motif (α2- α3), and a β hairpin motif (β5- β6). Furthermore, wing domains 1 and 2 as well as the long α-loop connecting α2 and α3 are highlighted. Wing domain 1 is the connecting turn between β5 and β6, whereas wing 2 is G48, which is located between β4 and α1. *B*, sequence and secondary structure of cToxR_1–134: β-strands are shown as *arrows*, α helices are shown as *cylinders*. Wing domains 1 and 2 as well as the α loop region are marked.

The structure of cToxR_1–134 reveals an OmpR-like wHTH fold (16), consisting of an N-terminal β sheet, a helix–turn–helix motif, and a β-hairpin wing (β1- β2- β3- β4- α1- α2- α3- β5- β6- β7) (Fig. 1). The four antiparallel N-terminal β strands are forming a five stranded β-sheet with β strand β7. This structural connection of the termini leads to a compact fold of the protein. The HTH motif is composed of positioning helix α2 and recognition helix α3. The structure of cToxR_1–134 reveals the formation of a 12 amino acids long loop linking the helices of the HTH motif, which is quite long for HTH motifs since they are usually comprised of 5 to 6 residues (43). The loop of cToxR_1–134 is referred to as α-loop since it is proposed to interact with the α-subunit of the RNAP (44) similar to wHTH protein OmpR (43). Following the HTH motif, strands β5–β6 are forming a hairpin motif, including wing domain 1 (Fig. 1).

### The soluble cytoplasmic domain of ToxR binds DNA

**Figure 2:**
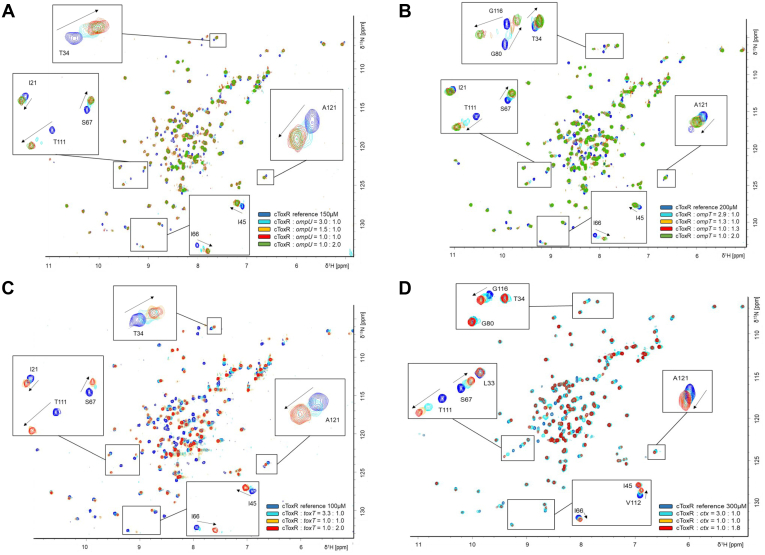
**cToxR_1-134 binds *V. cholerae* promotor regions.***A*–*D*, NMR titration experiments of ^15^N cToxR_1–134 with ds DNA oligos, present in *V. cholerae* promotor regions of ToxR affected genes (*ctx, ompU, ompT, toxT*). Base sequences are listed in Table 2 in the Experimental procedures section. CSPs reveal a general affinity of the cytoplasmic domain of ToxR to DNA. Zoomed-in regions display CSP for specific amino acids that are affected upon binding.

Guided by the structure of cToxR_1–134, we now investigated the details of its binding to different DNA motifs of *V. cholerae* promoter regions using chemical shift perturbation (CSP) experiments (Fig. 2). We confirmed that the missing C-terminal residues of cToxR-1–134 are not involved in DNA interactions (Fig. S5). To this end, the following double-stranded (ds) DNA oligos containing the described recognition site for ToxR (45, 46): *ompU*, *ompT*, *toxT*, and *ctx* (Tables 1 and 2) were titrated to ^15^N uniformly labeled cToxR_1–134 in different ratios. Amino acids located in the binding interface, interacting directly with the DNA, typically experience a stronger CSP than amino acids that are only indirectly affected, for instance, by structural changes upon binding.

**Table 1:** Calculation of the NMR-based and fluorescence anisotropy FA-based dissociation constants K_d_ of cToxR_1–134 to *toxT, ompT, ompU*, and *ctx* motifs and to a randomly selected DNA strand

NMR-based dissociation constant K_d_				
K_d_ cToxR-[μM]	*toxT*	*ompU*	*ompT*	*ctx*
T111	0.15 ± 4∗10^−9^	6.82 ± 1.07	14.21 ± 0.31	28.26 ± 5.73
L99	0.63 ± 1∗10^−8^	5.16 ± 0.46	14.78 ± 6.62	27.88 ± 3.01
	0.35	5.99	14.50	28.07

Fluorescence-anisotropy-based dissociation constant K_d_					
K_d_ cToxR-[μM]	*toxT*	*ompU*	*ompT*	*ctx*	*DNA*
	0.66 ± 0.1	5.49 ± 0.91	12.75 ± 1.27	9.71 ± 0.84	∗

Base sequences are listed in Table 2 in the Experimental procedures section. Fields marked with an asterisk could not be fitted.

**Table 2:** Base sequences of the ds DNA oligos of *V. cholerae* DNA motifs containing the ToxR binding site (*ctx, ompT, ompU, toxT*)

DNA oligos	Base sequence
*ctx* (coding strand) *ctx* (complementary strand)	5′- GAT TTT TGA TTT T -3′ 3′- AAA ATC AAA AAT C -5′
*ompT* (coding strand) *ompT* (complementary strand)	5′- TTT TAT GGT ATT TGA -3′ 3′- TCA AAT ACC ATA AAA -5′
*ompU* (coding strand) *ompU* (complementary strand)	5′- ATT TAT ATC ATT TTA -3′ 3′- TAA AAT GAT ATA AAT -5′
*toxT* (coding strand) *toxT* (complementary strand)	5′- CTC AAA AAA CAT AAA A -3′ 3′- TTT TAT GTT TTT TGA G -5′
random DNA (coding strand) random DNA (complementary strand)	5′- TAC GTA TTT ATA CAT -3′ 3′- ATG TAT AAA TAC GTA -5′

Additionally, a randomly selected DNA strand was tested having a similar AT content as previously mentioned oligos.

The titrations reveal similar amino acid contributions but different binding strengths to the added annealed DNA oligos (Fig. 2). These experiments suggest that the transmembrane and the periplasmic domain are not essential for ToxR to bind DNA. Furthermore, the results propose that the membrane attachment of ToxR is not crucial for the interaction process. Nevertheless, we cannot exclude that the presence of the domains and/or the membrane localization may alter ToxR DNA-binding specificity and strength.

### ToxR binds *toxT* motif with higher affinity compared with *ompU*, *ompT*, and *ctx*

Apparent dissociation constants were derived based on the CSP of Threonine 111 and Leucine 99 (Table 1 and Fig. S7). In Figure 2, the CSP of T111 after the addition of different DNA oligos is highlighted. T111 is located near wing domain 1 and forms part of β 5. L99 is forming the loop region located near the C-terminal end of the recognition helix α3 (Fig. 1). Additionally, we performed fluorescence anisotropy FA experiments with 5′ FITC modified oligos (see ‘Experimental procedures’) to confirm NMR-based dissociation constants. For FA experiments we also used a scrambled DNA strand having a similar AT content than the ToxR recognition site. The base sequences are listed in Table 2 in the Experimental procedures section.

The strongest binding (NMR derived K_d_: 0.35 μM, FA derived K_d_: 0.66 μM) is measured between cToxR_1–134 and *toxT* (Table 1 and Figs. S7 and S8). The role of ToxR in the activation of ToxT is probably to catch the DNA and bring it near to the membrane so the main activator TcpP can interact with the *toxT* operon (26). TcpP binds DNA with low affinity and therefore needs ToxR as a coactivator to enable its activation process (19, 26, 29, 30). The role of ToxR as a “DNA-catcher” for *toxT* transcription activation therefore implies a strong affinity for its binding site on the *toxT* operon.

Furthermore, cToxR_1–134 seems to bind *ompU* more efficiently than *ompT*, which is repressed by ToxR (Table 1 and Figs. S7 and S8). cToxR_1–134 binds also to the scrambled DNA strand (Fig. S8), which has a similar AT content compared with the predicted ToxR binding sites. Nevertheless, binding occurs with low affinity and seems to be unspecific. Since we were not able to reach saturation in FA experiments, we could not calculate a dissociation constant (Table 1).

The calculated NMR and FA-based dissociation constants between cTocR_1–134 and *ctx* reveal slightly different outcomes. In the NMR experiments we calculated a K_d_ of 28 μM, whereas FA experiments show a lower value of 9.7 μM (Table 1). In the case of *ctx*, ToxR is not the natural activator, only when overexpressed it can overcome the lower binding affinity and activate the transcription (8, 32, 33, 34, 35).

ToxR obviously binds to DNA generally with weak affinity, which is typically observed for DNA-binding proteins, but with highly increased affinity for specific, physiologically targeted base sequences. According to the proposed consensus binding site of ToxR, namely “tox-box,” which is determined as “TNAAA-N_5_-TNAAA,” ToxR seems to prefer AT-rich DNA sequences (20). Nevertheless, the correct consensus sequence seems to be essential for a tight interaction. Using a scrambled DNA oligo with a similar AT-content reveals only weak interactions with cToxR_1–134 (Table 1 and Fig. S8). Finally, the presence of the other domains as well as the membrane localization may further influence its binding behavior and explain the different mechanism by which ToxR can influence the transcription of numerous genes.

### The ToxR-DNA interaction is established *via* the HTH motif and wing 1

**Figure 3:**
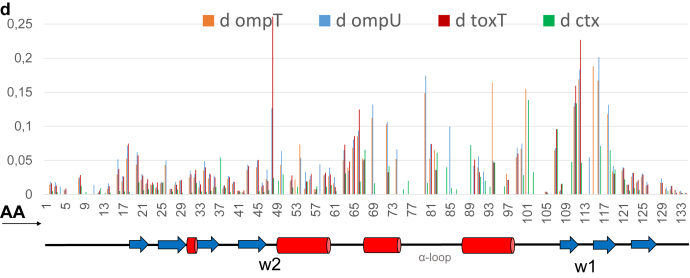
**Analysis of the chemical shift changes of**^**1**^**H-**^**15**^**N signals of cToxR_1–134.** The x-axis represents the amino acid sequence. For each residue, the degree of the chemical shift changes was calculated represented by the d-value (see Experimental procedures). Four titrations were performed using ds oligos from promotor regions of: *ompT* (*orange*), *ompU* (*blue*), *toxT* (*red*), and *ctx* (*green*). Base sequences are listed in Table 2 in the Experimental procedures section. The highest chemical shift changes/d-values could be detected around wing domain 1, recognition helix 3 and G48 (=wing 2). These regions are mostly affected upon DNA binding, proposing an essential function in the interaction event.

**Figure 4:**
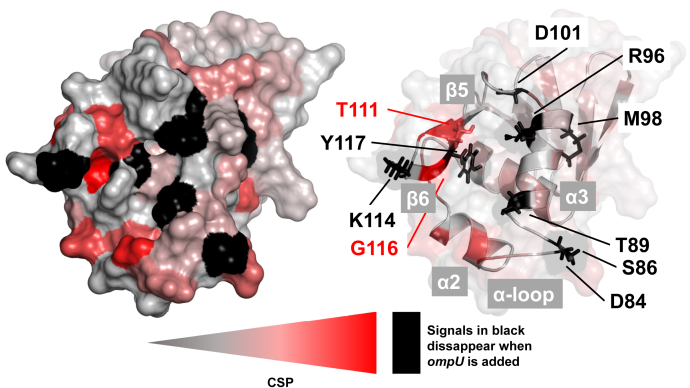
**Chemical shift changes of cToxR_1–134 upon binding of *ompU* highlighted on the structure according to a color gradient.** Residues coloured in *red* experience a strong effect on their chemical shift. Residues colored in *black* disappear upon ompU addition. *Red* and *black colored residues* appear mostly in the helix–turn–helix motif (α2 and α3) and the C-terminal wing domain 1 (β5 and β6), thus forming the binding site of the DNA. Some residues are shown in *sticks* and colored according to the described scheme.

The CSPs of cToxR_1–134 (derived from data presented in Fig. 2) were analyzed as described in Experimental procedures by calculating the d-value representing the degree of change for each signal (45) (Fig. 3). Peaks located in the crowded regions of the spectra could not be included in the calculation due to severe overlap. Signals that are broadened beyond detection upon addition of the DNA oligos (intermediate exchange interaction) are discussed separately. Because ToxR acts as a direct activator for *ompU* transcription (21, 22), we decided to select the outcomes of the *ompU* NMR titration experiments for the location of the DNA-binding site on the structure of cToxR_1–134. Figure 4 displays the structure of cToxR_1–134 colored according to the calculated d-values of each residue (45). Additionally, residues disappearing when bound to *ompU* are highlighted in black.

The residues T111 and G116, which are conserved in wHTH proteins (16), are located in the C-terminal β hairpin region of cToxR (Fig. 4) and reveal a severe change of their chemical shift in all four titration experiments (Fig. 3), proposing an important role in the DNA interaction. Mutation in each of these amino acids (T111K, T111R or G116S) results in a loss of function, *i.e.*, that ToxR is no longer capable of activating *ompU* and *toxT*, as well as binding to the *toxT* promoter (44).

The two residues K114 (forms part of wing 1) and Y117 (forms part of β strand 6) are binding in the intermediate exchange regime, meaning that they disappear in all four titration experiments upon DNA addition due to signal broadening, thereby indicating their important contribution to DNA binding (Fig. 4). Several residues showed intermediate exchange binding with *ompU*, *ompT*, or *toxT*, but fast exchange in the presence of *ctx* (Fig. 3). Those residues are: D84 and S86 (both located in the C-terminal region of the α-loop, which is close to recognition helix 3); T89, R96, and M98 (located in recognition helix 3) and D101 (located in the loop following recognition helix 3) (Fig. 4). The previously mentioned residue R96 is part of the C-terminal end of the recognition helix 3 and is highly conserved in wHTH proteins due to its crucial function in the establishment of the DNA–protein complex (16).

Residues that chemical shifts are only slightly or not at all affected by the binding are most likely not directly involved in the interaction (Fig. 3). Those residues are mostly located in helix 1 and the five stranded β-sheet, formed by the four N-terminal β strands (β1- β4) and the C-terminal β strand (β7). G49 shows distinctive chemical shift changes (Fig. 3). It connects β strand 4 and α helix 1 and is proposed to be wing domain 2 (Fig. 1) (16). Since it is not located near the binding site, it might experience conformational changes or allosteric effects upon DNA binding.

Taken together, residues that seem to be directly interacting with the DNA are mostly found in the HTH motif and the C-terminal β hairpin including wing 1 (Figs. 3 and 4).

Residues K114 and Y117, located in the wing domain 1 region, are shown in black since their peaks disappear in all four titration experiments upon addition of DNA. Those residues could be essential for general binding of DNA. T111 and G116, shown in red sticks are part of β5 and β6 respectively, and experience a strong change of their chemical shift upon addition of each DNA motif.

D84 and S86 (C-terminal region of the α-loop); T89, R96, and M98 (recognition helix 3); and D101 (loop following recognition helix 3) disappear when ompU, ompT, or toxT motifs are added; when ctx is added, they experience a change of their chemical shift.

### ToxR contacts the major and the minor groove of the DNA with its α-loop exposed to the solvent

**Figure 5:**
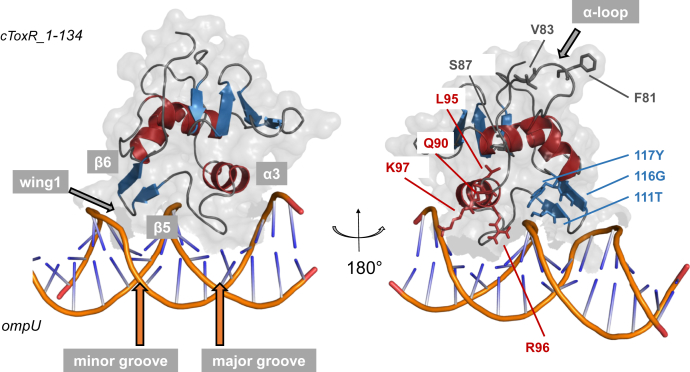
**NMR guided HADDOCK model of cToxR_1–134-*ompU* complex.** The recognition helix 3 as well as its following loop is binding into the major groove of the dsDNA, whereas the C-terminal hairpin including wing 1 is positioned in the minor groove. The C-terminal residues of the α-loop are stabilizing the positioning of the recognition helix 3 by forming contacts with the DNA backbone. The rest of the long α-loop is accessible for interactions with, *e.g.*, the RNAP and is exposed to the surface of cToxR_1–134.

We next determined a structural model of the cToxR_1–134-*ompU* complex. The HADDOCK server (38, 39) allowed us to use the experimental CSPs to perform data-driven docking. In the lowest-energy conformation of the data-driven docking process cToxR_1–134 binds simultaneously to the minor and the major groove of double-stranded DNA (Fig. 5). The long α-loop, connecting helices 2 and 3, is mostly exposed to the solvent where it is accessible for interactions with, *e.g.*, the α-subunit of the RNAP (Fig. 5). Mutational experiments propose that residues F81 and V83, located in the α-loop of ToxR, may be essential for the activation of the RNAP at the *ompU* promoter (44). S87, which is close to recognition helix 3, seems to be crucial for the interaction with DNA since a mutation of S87 to alanine leads to severe loss of ToxR DNA binding ability and activation of *toxT* and *ompU* (44).

#### ToxR recognition helix and its following nine residues long loop are placed in the major groove of the DNA

The amphipathic recognition helix α3 contacts the bases of the major groove with its surface exposed polar region (Fig. 5). The amino acid composition of the solvent accessible region of α3 is important for specific DNA base interactions and is therefore (except of a conserved arginine R96) different for each wHTH protein (16). The polar surface of the recognition helix of cToxR_1–134 consists of T89, Q90, S93, T94, R96, and K97. The hydrophobic residues of the recognition helix contribute to the hydrophobic core of the protein and include a conserved leucine residue L95 (16) (Fig. 5). Indeed, mutational studies of cToxR reveal that mutants Q90R and L95P are defective for *toxT* promoter binding and activation of *ompU* and *toxT* (44).

In addition, the nine residues long loop following recognition helix 3 and connecting it to the C-terminal β hairpin motif is also arranged in the major groove of the DNA (Fig. 5). This region also shows significant changes of the chemical shift (Fig. 3), thereby proposing a putative important function in the interaction process.

#### The β hairpin and wing 1 of ToxR bind into the minor groove

The β hairpin wing region contacts the minor groove of the DNA (Fig. 5) and contains three conserved residues (T111, G116, Y117). T111 of ToxR is located in β strand 5, whereas G116 and Y117 are part of β strand 6. Mutation of threonine to lysine or arginine and mutant G116S result in a loss of ToxR ability to activate transcription of *ompU* and *toxT*, as well as binding to the promoter of *toxT* (29, 44). There are indications that the wing region of cToxR may be important for the interaction with TcpP and therefore for the activation of the *toxT* genes (19, 29). The mutant P113L reveals reduced levels of interaction with TcpP and is defective of ToxT expression (29).

### The isolated cytoplasmic domains of ToxR and TcpP do not interact in solution

Additionally, we performed NMR chemical shift mapping experiments with the soluble cytoplasmic domains of ToxR and TcpP, which did not show changes of the chemical shift (Fig. S6). Thus, there is no binding event of the isolated cytoplasmic domains of *toxT* main activator TcpP and coactivator ToxR under the applied conditions.

Because ToxR and TcpP interaction is proposed to be crucial for *toxT* transcription activation, we suggest that the presence of the full-length proteins and/or the membrane attachment of the proteins are likely needed to achieve effective binding. Alternatively, other factors might be involved in the establishment of this protein–protein interaction. These results support the outcome of studies published by Crawford *et al.* (15), which suggest that membrane localization of ToxR is essential for the interaction between ToxR and TcpP but is not required for DNA binding and ToxR-dependent transcription activation.

### Wing domain 1 of cToxR shows high sequence and structural similarity to “ToxR-like” wHTH protein CadC

The residues establishing the interaction with the DNA are conserved among wHTH proteins (16). They are mostly located nearby the wing domains and the recognition helix 3 (Fig. S9). In ToxR, the conserved residue E51 is part of the N-terminal region of helix 1 but is structurally close to recognition helix 3 (Fig. S10*A*). E51 seems to stabilize the positioning of DNA-binding helix 3 by forming contacts to the apolar residues of the amphipathic helix. Its polar carboxylic acid group is pointing toward the solvent. Interestingly, CadC, a “ToxR-like” transcription factor from *E. coli*, contains a hydrophobic leucine residue at this position (37) (Fig. S10*A*).

The conserved L95 and R96 residues are part of the recognition helix 3 in cToxR (Fig. 5). L95 is involved in hydrophobic interactions stabilizing the position of the helix and contributing to the hydrophobic core. The polar R96 is exposed to the surface and essential for the binding to DNA. Its positively charged side chain forms ionic interactions with the phosphate sugar backbone of the DNA.

**Figure 6:**
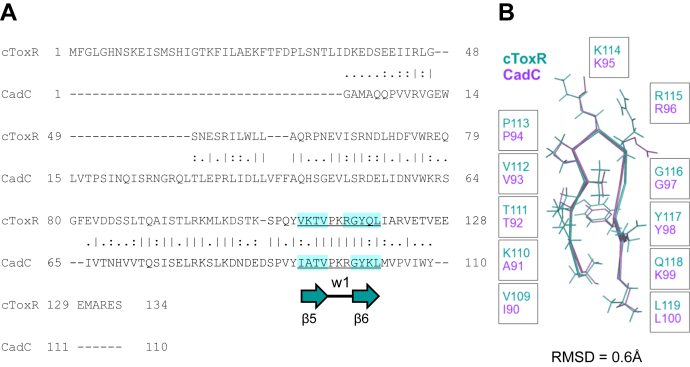
**cToxR and CadC reveal significant structural and sequential similarities in wing domain 1.***A*, pairwise sequence alignment (EMBOS Needle (67)) of “ToxR-like” wHTH proteins cToxR and CadC (*E. coli*). The highlighted region is forming a β-hairpin motif including wing domain 1 as a connecting turn. This region is highly important for the DNA interaction and shows a high sequence similarity between the proteins. Comparison with other wHTH proteins does not show a significant sequence similarity in the described region. *B*, structural alignment of the β-hairpin motif of Cadc and cToxR reveals an all-atom RMSD of 0.6, confirming a high structural similarity.

T111, G116, and Y117 are mostly solvent exposed and located nearby wing 1, which is contacting the minor groove of the DNA (Fig. 5). Interestingly, the sequence alignment between the “ToxR-like” receptors, cToxR and CadC, reveals a high sequence identity in the β hairpin region (Fig. 6). Other wHTH proteins do not show a significant sequence similarity in this region, apart from the three mentioned conserved residues. So far, the structures of CadC and cToxR are the only “ToxR-like” protein structures available. It would be interesting to compare our results with future “ToxR-like” proteins to confirm if the described similarities are general properties of this subfamily of wHTH proteins.

### “ToxR-like” receptors ToxR and CadC share a common topology of their DNA-binding domain

**Figure 7:**
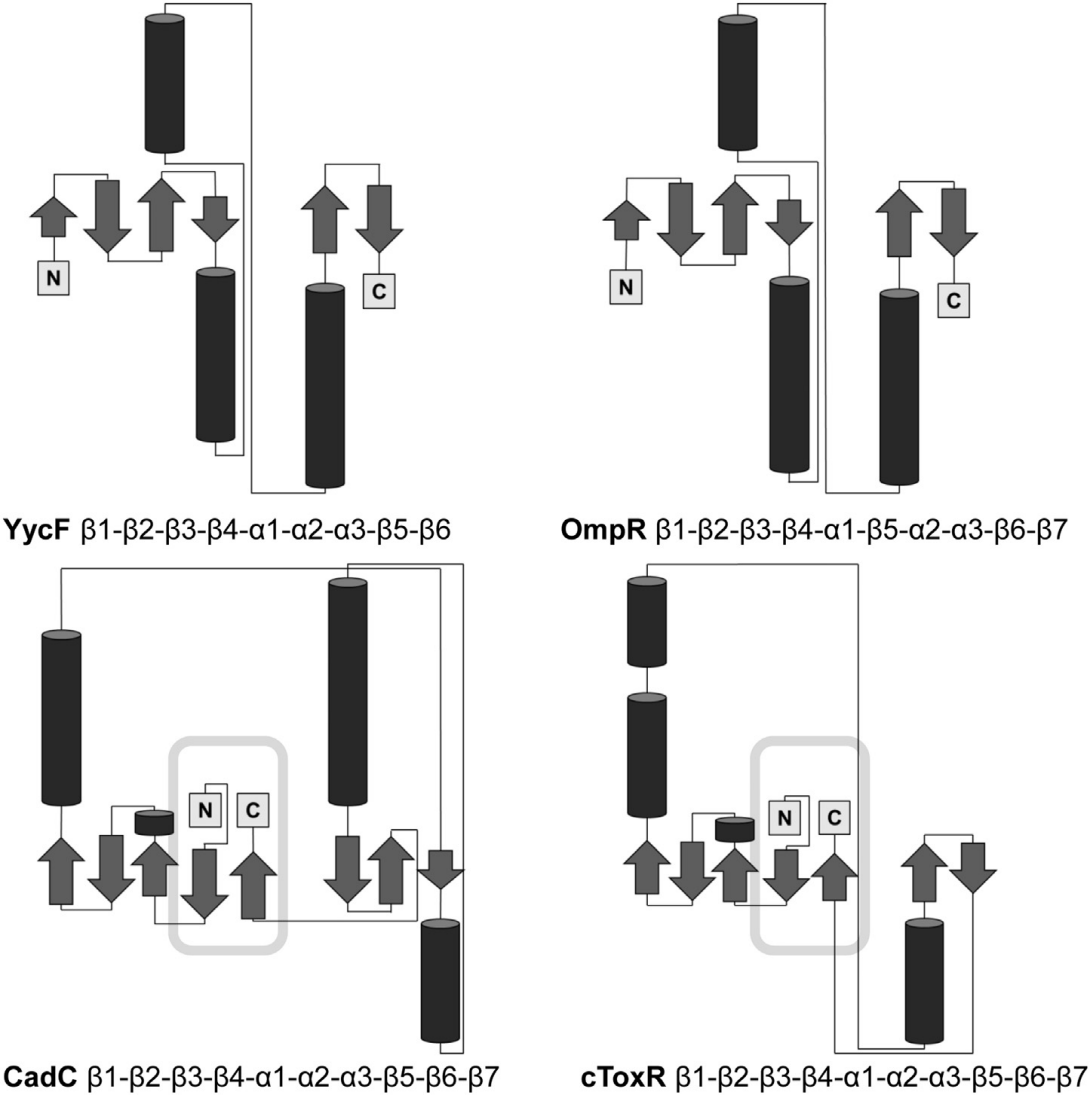
**Topology diagrams of the DNA-binding domains of four wHTH proteins: YycF (*Bacillus subtilis*, pdb code:****1d1c****), OmpR (*E. coli*, pdb code:****1opc****), CadC (*E. coli*, pdb code:****5ju7****) and ToxR.** CadC and ToxR are forming a subgroup of this structural protein family, referred to as “ToxR-like” receptors. The five stranded β sheet formed by four N-terminal β strands and one C-terminal strand is only present in “ToxR-like” proteins.

The structure of cToxR_1–134 reveals an OmpR-like wHTH fold, consisting of an N-terminal β sheet, a helix–turn–helix motif, and a β-hairpin wing (β1- β2- β3- β4- α1- α2- α3- β5- β6- β7). In contrast to the four stranded β-sheet present in OmpR, ToxR forms a five stranded β-sheet comprised by β strand β7, which is not present in OmpR, and four antiparallel N-terminal β strands (β1–β4) (Fig. 7 and Fig. S10*B*). This structural connection of the termini leads to a compact fold of the protein. Interestingly, this conformation could also be found in the structure of the DNA-binding domain of the *E. coli* transmembrane regulator CadC, which also belongs to the wHTH subgroup of “ToxR-like” receptors (37, 47). “ToxR-like” receptors share the property of having three domains with the middle one spanning through the inner membrane (47). The periplasmic sensory domain is connected by a transmembrane single helix to the cytoplasmic wHTH containing DNA-binding domain. The signal transduction is achieved without chemical modification (47).

## Conclusion

The inner membrane spanning transcription regulator ToxR of *V. cholerae* represents a complex system involved not only in virulence-associated processes. To further clarify the mechanisms of this versatile regulator, we present structural and functional studies on its soluble cytoplasmic DNA-binding domain (cToxR_1–134).

The determined structure of cToxR_1–134 confirms the presence of a wHTH motif and additionally revealed unexpected structural features, *e.g.*, the presence of an additional β strand at the C-terminus forming contacts with the N-terminal β-sheet. The presented structural analysis could be useful for the comparison to future structures of “ToxR-like” receptors and thus for the determination of common features of this yet poorly defined subgroup of the wHTH protein family.

Our data furthermore provide direct evidence for the binding of the isolated cytoplasmic domain of ToxR to DNA and deliver detailed insights into this regulatory binding process. In summary, the protein–DNA complex formation is achieved by binding of ToxR to the minor and the major groove of double-stranded DNA with its wing domain 1 and its recognition helix region, respectively.

Analysis of the CSP of cToxR_1–134 residues upon addition of different *V. cholerae* DNA motifs (*ompU*, *ompT*, *toxT*, *ctx*) reveals the strongest affinity of cToxR_1–134 to the *toxT* motif. Regarding the activation of *toxT*, ToxR is proposed to act as a cofactor bringing the DNA to the membrane to facilitate binding of the inner membrane spanning activator TcpP. A strong binding of ToxR to *toxT* could be therefore mandatory to activate *toxT* trancription.

Protein–protein interaction between inner membrane proteins ToxR and TcpP is described to be essential for virulence-associated *toxT* activation in *V. cholerae*. Our NMR titration experiments with the cytoplasmic domains of ToxR and TcpP support the current theory that although membrane attacement is not required for ToxR to bind DNA, it is probably essential for its interaction with TcpP (15).

## Experimental procedures

### Cloning, production, and purification of recombinant proteins

The chemically synthesized, *E. coli* codon-optimized, gene (purchased from ATG biosynthetics) of cToxR_1–134 (Fig. S1) was inserted into a pET45b vector using standard procedures, containing an N-terminal His_6_ purification tag. Protein expression was achieved using *E. coli* BL21 DE3, grown in minimal media M9 containing 100 mg/ml ampicillin and enriched with either 1 g/l ^15^NH_4_Cl for NMR titration studies or 1 g/l ^15^NH_4_Cl and 2 g/l ^13^C-glucose for NMR assignments. Cells were grown to an OD_600_ of 0.6 to 0.8 at 37 °C, induced with 1 mM Isopropyl-β-D-thiogalactopyranosid IPTG and grown overnight at 20 °C before harvesting by centrifugation. The pellet was resuspended in a sodium phosphate buffer (10 mM Na_2_HPO_4_, 300 mM NaCl, 10 mM imidazole 0.02% NaN_3_ pH 8) and sonicated. Lysate was centrifuged, the supernatant was applied on a 5 ml His-Trap Nickel HP column and eluted with a linear imidazole gradient. The final purification step was achieved by size exclusion chromatography using a HiLoad 26/600 Superdex 75 pg column. The fractions containing cToxR_1–134 were buffer exchanged to NMR buffer (50 mM Na_2_HPO_4_, 100 mM NaCl pH 6.5).

The ORF of the cytoplasmic domain of TcpP was codon-optimized for *E. coli* expression and the chemically synthesized gene of cTcpP_1–142 (purchased from ATG biosynthetics) was inserted into a pET-Z2 vector containing an N-terminal His6 purification tag, an Z2 solubility tag, and a TEV cleavage site (48) using standard procedures. *E. coli* BL21 DE3 strain was used for cTcpP expression, the transformed cells were grown in minimal media M9 containing 50 mg/ml kanamycin in presence of 1.5 g/l ^15^NH_4_Cl for NMR titration studies or 1 g/l ^15^NH_4_Cl and 2 g/l ^13^C-glucose for NMR assignments. Upon growth up to OD_600_ of 0.6 to 0.8 at 37 °C, protein expression was induced with 1 mM Isopropyl-β-D-thiogalactopyranosid (IPTG). Expression was performed overnight at 16 °C. Cells were harvested by centrifugation and the pellet was resuspended in a Tris buffer pH 8.0 in the presence of 150 mM NaCl, 10 mM Imidazol, 1 mM MgCl_2_ and 1 mM MnSO_4_, 0.1 mg/ml of DNase I (SIGMA) and sonicated in 10 s pulses with 10 s intervals. Cell lysate was centrifuged, and the supernatant was applied to a gravity column packed with 3 ml Ni-NTA Agarose resin (QIAGEN). After two washing steps, the bound protein was eluted with 10 ml Tris buffer pH8.0 containing 150 mM NaCl and 350 mM Imidazol. The eluted protein was dialyzed overnight in Tris buffer pH 8.0 in the presence of 1 mg TEV protease and 5 mM BME, the dialyzed protein was loaded in a gravity column as described above, and the flow through was concentrated and loaded into a HiLoad 26/600 Superdex 75 pg column equilibrated with Phosphate buffer pH 6.5 and 150 mM NaCl. The monomeric cTcpP was recovered from the respective fractions according to the expected size, and the protein was finally concentrated up to 0.8 to 1.0 mM before NMR experiments.

### NMR experiments

All spectra were recorded at 298K on a Bruker Avance III 700 MHz spectrometer, equipped with a 5 mm cryogenically cooled TCI probehead in 90% of NMR buffer (50 mM Na_2_HPO_4_, 100 mM NaCl pH 6.5) and 10% (v/v) D_2_O. Spectra for backbone and side chain assignment were recorded with a 400 μM cToxR_1–134 sample. For the assignment of the backbone resonances, standard triple resonance experiments were used: HNCO (49, 50), HN(CA)CO (50, 51), HNCACB (52, 53), HN(CO)CA (49, 50), HNCA (49, 50, 54), HN(CA)CO (50, 51), and a ^15^N HSQC experiment (55). For side-chain resonance assignments we used: HCCH-TOCSY (56, 57, 58), H(CCCO)NH (59, 60), and (H)C(CCO)NH (59, 60). Spectra were processed using NMRPipe (61). The backbone and side-chain resonance assignments (Table S1) were carried out with CcpNMR 2.4.1 (62). NMR Protein DNA titrations were carried out under the same buffer conditions described before. Molecular images were created using PyMOL (Delano Scientific, (http://www.pymol.org/pymol). Secondary structure predictions were done using backbone assignments and TALOS+ (40).

### CS Rosetta

Structural models of cToxR were obtained using the well-established CS-Rosetta (41, 42) protocol. Using talos predictions (40) (Fig. S3), the flexible termini were excluded from the calculation and the following data preparation and structure prediction steps were performed on residues I16-E128 of cToxR: Backbone and sidechain chemical shift data was obtained as described in the previous sections and was used for Rosetta fragment selection. From the observed set of NOE contacts (Table S2), distance restraints were created for the full atom as well as the centroid mode of the Rosetta framework. Using these input data sets, an ensemble of 100.000 structures were computed by running the Abinitio Rosetta protocol. Subsequently, all structures of the obtained ensemble were ranked based on a combined score (sum of the Rosetta score score13_env_hb, the chemical shift score, and the atom pair constraint score) and the RMSD to each of the ten best-ranked models were computed (plots for best ranked model are shown in Fig. S4). The obtained score-*versus*-RMSD plots show a clear funnel toward the best-scored model, indicating that the CS-Rosetta structure prediction has converged. Consequently, the best scored model was used for further structure prediction as described in the main text.

### Preparation of double-stranded ds DNA strands

Ds DNA oligos were purchased by Vienna Biocenter VBC Genomics and dissolved in water to yield 1 mM stock solutions. The double-stranded DNA oligos (except of the random strand) are motifs from *V. cholerae* operons proposed to contain ToxR binding site “TNAAA-N_5_-TNAAA” (15, 20, 26, 63). The base sequences are listed in Table 2.

### Analysis of chemical shift perturbations

To detect the interface of the interaction, we used formula (I.) described in the publication by Williamson (45).

(1) $$\text{d}=\sqrt{\frac{1}{2}\left[\text{δ}{\text{H}}^{2}+\left(\text{α∗δN}\right)ˆ2\right]}$$

δH/δN…chemicalshiftchanges[ppm]

α…scalingfactorαα=0.14,exceptforglycinsα=0.2

The calculated d-values give information about the degree of change of the chemical shift after the DNA addition. Peaks that disappear after addition of DNA or peaks located in the crowded middle region of the spectra may also be influenced by the binding but cannot be included in the calculation. To determine which residues show a significant change of the chemical shift, the standard deviation sigma was calculated as described by Williamson (45). Residues that show a higher d-value than sigma are affected upon binding.

### Calculation of the cToxR_1–134-*ompU* HADDOCK model

We used the lowest energy model of the CS-ROSETTA calculation as a starting structure for the protein–DNA complex. First, we used the NMRPipe package (61) to add hydrogens to the structure. Then, we performed a short molecular dynamics simulation in order to remove potential steric clashes from the hydrogen addition. For this, we fixed backbone atoms that showed secondary structure elements and ran the simulation for 1 ps using the XPLOR-NIH package (64). We generated ten structures and the lowest-energy conformation was used for further docking. To create a structural model for the DNA sequence, we used the 3D-DART server (65) to generate the B-helix form of the sequence 5′-ATTTATATCATTTTA-3′ together with its reverse complement. To generate a protein-DNA structural model, we employed the HADDOCK 2.2 server (38) using the structures mentioned above. The NMR-titration data was used to drive the docking process and the following residues with the strongest chemical shift changes (>0.08) were used to define the active protein interactions: G58, I76, H82, G90, F91, V122, R125, G126. Passive residues were automatically determined. On the DNA side, we defined the central three bases as active residues and the passive residues to be automatically defined. For data interpretation and figure generation, we used the cluster with the lowest overall HADDOCK-score and the number 1 best structure.

### Calculation of dissociation constants by NMR

In order to calculate Kd values, CSPs extracted from ^1^H-^15^N HSQC were weighted (Equation 2) (45) and a scaling factor of 0.14 included. Subsequently, the weighted shifts were fitted in equation (Equation 3) (45), where Δδ_obs is the weighted shift, [P_t] is total protein concentration, and [L_t] is ligand concentration using data analysis software QtiPlot (version 5.9.8).

(2) $$\Delta \text{δ}\_\text{obs}=\sqrt{\left(1/2\left[〖\text{δ\_Hˆ}2\;+\;\left(\text{α.δ\_N}\right)ˆ2\right]\right)}$$

(3) $$\text{Δδ\_obs}=\left(\text{Δδ\_max}\left\{\left(\text{n}\left[\text{P\_t}\right]+\left[\text{L\_t}\right]+\text{K\_t}\right)-\sqrt{\left(\left(\left[\text{P\_t}\right]+\left[\text{L\_t}\right]+\text{K\_t}\right)ˆ2-4\left[\text{P\_t}\right]\left[\text{L\_t}\right]\right)}\right\}\right)/2\left[\text{P\_t}\right]$$

### Calculation of dissociation constants by fluorescence anisotropy

5′- FITC-labeled DNA oligos (see Table 2) were purchased by Eurofins. The interaction between dsDNA and cToxR was measured in NMR buffer (50 mM Na_2_HPO_4_, 100 mM NaCl pH 6.5). The fluorescence anisotropy of a 100 nM FITC-labeled dsDNA solution was measured at 25 °C on a Jasco FP-6500 spectrofluorimeter (Jasco Inc), equipped with excitation and emission polarizers, at an emission wavelength of 525 nm upon excitation at 495 nm. Slit widths were 5 nm and 10 nm for excitation and emission, respectively. The fluorescence anisotropy is defined as shown in (Equation 4) (66), where I_VV_ is the fluorescence intensity recorded with excitation and emission polarizers in vertical positions, and I_VH_ is the fluorescence intensity recorded with the emission polarizer aligned in a horizontal position. The G factor is the ratio of the sensitivities of the detection system for vertically and horizontally polarized light G = I_HV_/I_HH_.

(4) $$\text{r}=\left({\text{I}}_{\text{VV}}-\text{G}\times {\text{I}}_{\text{VH}}\right)/\left({\text{I}}_{\text{VV}}+2\text{G}\times {\text{I}}_{\text{VH}}\right);-0.2\leq \text{r}\leq 0.4$$

FITC-dsDNAs were titrated against increasing amounts of cToxR. For each point, the anisotropy was recorded over 30 s and the mean r values for each measurement were used. Anisotropy changes were fitted to the following equation (Equation 5) by using the Levenberg–Marquardt algorithm where r is the observed anisotropy, Δr_max_ is the maximal anisotropy change, and K_D_ is the dissociation constant. *dsDNA* indicates any DNA oligo used in the present study.

(5) $$r=\Delta r_{max}\frac{K_{D}+\left[cToxR\right]+\left[dsDNA\right]-\sqrt{\left(K_{D}+\left[cToxR\right]+{\left[dsDNA\right]}^{2}\right)-4\left[cToxR\right]\left[dsDNA\right]}}{2\left[cToxR\right]}$$

### NMR interaction experiment between cytoplasmic domains of ToxR and TcpP

The proteins were purified as described above. cToxR was ^15^N-labeled and the cytoplasmic domain of unlabeled TcpP was added to the sample as followed: 1:1, 1:2, 1:3. Both proteins were dialyzed in the same NMR buffer (50 mM Na_2_HPO_4_, 100 mM NaCl pH 6.5). Experiments were recorded at 298K.

## Data availability

Structural data was deposited at the Protein Data Bank and the Biological Magnetic Resonance Data Bank available at following accession codes: PDB ID 7NMB, BMRB ID 34606. The data will be released upon publication.

## Supporting information

This article contains supporting information (40, 67).

## Conflict of interest

The authors declare that they have no conflicts of interest with the contents of this article.
