# Supplementary material for: Structural and DNA-binding properties of the cytoplasmic domain of Vibrio cholerae transcription factor ToxR
Source: J Biol Chem. 2021 Sep 4;297(4):101167. doi: 10.1016/j.jbc.2021.101167 (PMC8517210; doi:10.1016/j.jbc.2021.101167)
Supplement: Supplemental Figures S1–S10 and Tables S1, S2 [file mmc1.pdf]

Supporting Information

Structural and DNA binding properties of the cytoplasmic domain of *Vibrio cholerae* transcription factor ToxR

Nina Gubensäk[1,2]*, Evelyne Schrank[1], Christoph Hartlmüller[7], Christoph Göbl[10], Fabio S. Falsone[1,11], Walter Becker[1,9], Gabriel E. Wagner[1,3], Sergio Pulido[1], N. Helge Meyer[1,8], Tea Pavkov-Keller[2,4,5], Tobias Madl[4,6], Joachim Reidl[2,4,5] and Klaus Zangger[1,4,5*]

[1] Institute of Chemistry / Organic and Bioorganic Chemistry, University of Graz, A-8010 Graz, Austria

[2] Institute of Molecular Biosciences, University of Graz, A-8010 Graz, Austria

[3] Diagnostic and Research Institute of Hygiene, Microbiology and Environmental Medicine, Medical University of Graz, 8010 A-Graz, Austria

[4] BioTechMed-Graz, Graz, Austria

[5] Field of Excellence BioHealth – University of Graz, Graz, Austria

[6] Gottfried Schatz Research Center for Cell Signaling, Metabolism and Aging, Institute of Molecular Biology & Biochemistry, Medical University of Graz, 8010 Graz, Austria

[7] Center for Integrated Protein Science Munich (CIPSM) at the Department of Chemistry, Technische Universität München, 87548 Garching, Germany

[8] Division of Experimental Allergology and Immunodermatology, University of Oldenburg, 26129 Oldenburg, Germany

[9] Department of Medical Biochemistry and Biophysics, Karolinska Institute, 171 77 Solna, Sweden

[10] Department of Pathology and Biomedical Science, University of Otago Christchurch, Christchurch 8140, New Zealand

[11] Institute of Pharmaceutical Sciences / Pharmaceutical Technology and Biopharmacy, University of Graz, A-8010 Graz, Austria

* To whom correspondence should be addressed.

Email: klaus.zangger@uni-graz.at

Tel: ++43 316 380-8673

Fax: ++43 316 380-9840

Correspondence may also be addressed to

Email: nina.gubensaek@uni-graz.at

Tel: ++43 316 380-5340

Fax: ++43 316 380-9840

Table of Contents:

- **Figure S1** Display of three membrane spanning domains of full-length ToxR, and the truncated His6 tagged construct of the cytoplasmic domain, referred to as cToxR_1-134, used for the experiments mentioned in this publication.
- **Table S1** Statistics of the NMR assignments of cToxR_1-134.
- **Figure S2** Amino acid sequence of cToxR_1-134. Underlined residues could not be assigned in the 2D ^{15}N-^{1}H HSQC.
- **Table S2** List of manually assigned NOEs of cToxR_1-134 characterized by their distance in the amino acid sequence.
- **Figure S3** Talos+ (Shen et al. 2009a) secondary structure prediction of cToxR_1-134 using NMR chemical shift information.
- **Figure S4** Scores of the 100.000 structure models obtained from CS-Rosetta.
- **Figure S5** ^{15}N-HSQC NMR experiments with full-length FL cToxR before (dark blue) and after the addition of *ctx* DNA (orange).
- **Figure S6** ^{15}N-HSQC experiment with the cytoplasmic domains of ToxR (blue) and TcpP (red).
- **Figure S7** Determination of dissociation constants between cToxR_1-134 and double-stranded DNA motifs from *V. cholerae* operons by NMR: *ompU, ompT, toxT* and *ctx*.
- **Figure S8** Determination of dissociation constants between cToxR_1-134 and double-stranded DNA motifs from *V. cholerae* operons by fluorescence anisotropy: *ompU, ompT, toxT* and *ctx*, as well as a randomly selected DNA strand.
- **Figure S9** Multiple sequence alignment (Clustal Omega (Madeira et al. 2019)) between wHTH proteins cToxR, cYycF (*Bacillus subtilis*, PDB code: 2d1v), cOmpR (*E. coli*, pdb code: 1opc) and CadC (*E. coli*, pdb code: 5ju7).
- **Figure S10** A: Structural overlay of ‘ToxR-like’ proteins cToxR_1-134 (red) and CadC (beige). B: Structural overlay of wHTH proteins cToxR_1-134 (red) and OmpR (turquois).

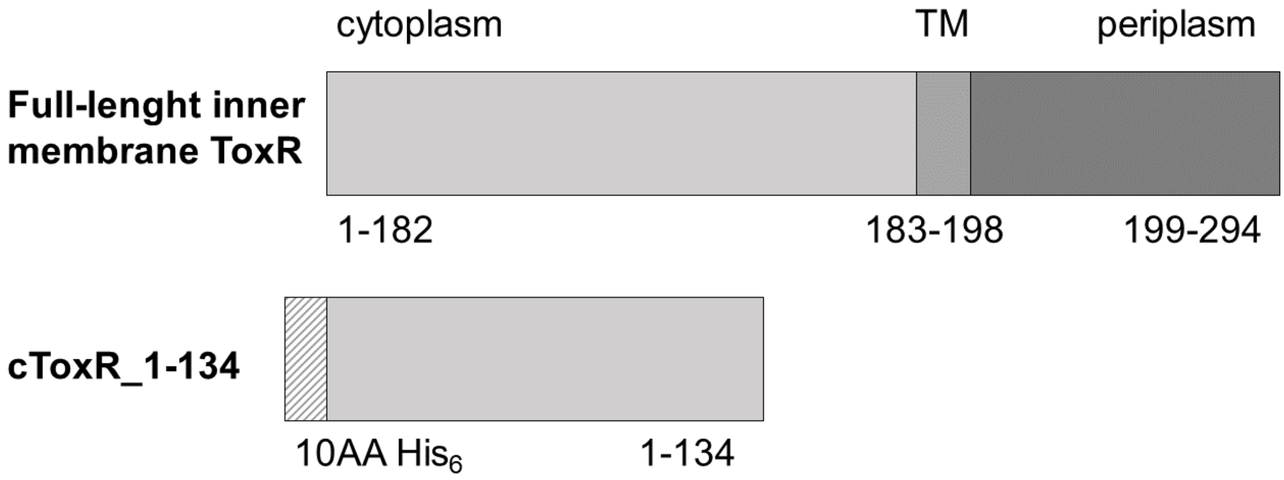

Supplementary Figure 1 Display of three membrane spanning domains of full-length ToxR, and the truncated His6 tagged construct of the cytoplasmic domain, referred to as cToxR_1-134, used for the experiments mentioned in this publication.

Supplementary Table 1 Statistics of the NMR assignments of cToxR_1-134.

Category	Available	Assigned	% Assigned
Backbone NH	266	244	92 %
All residues	135	127	94 %
Element H	826	516	63 %
Element C	668	449	67 %

```
         10         20         30         40         50         60
MFGLGHNSKE ISMSHIGTKF ILAEKFTFDP LSNTLIDKED SEEIIRLGSN ESRILWLLAQ
         70         80         90        100        110        120
RPNEVISRND LHDFVWREQG FEVDDSSLTQ AIST LRKMLK DSTKSPQYVK TVPKRGYQLI
        130
ARVETVEEEM      ARES
```

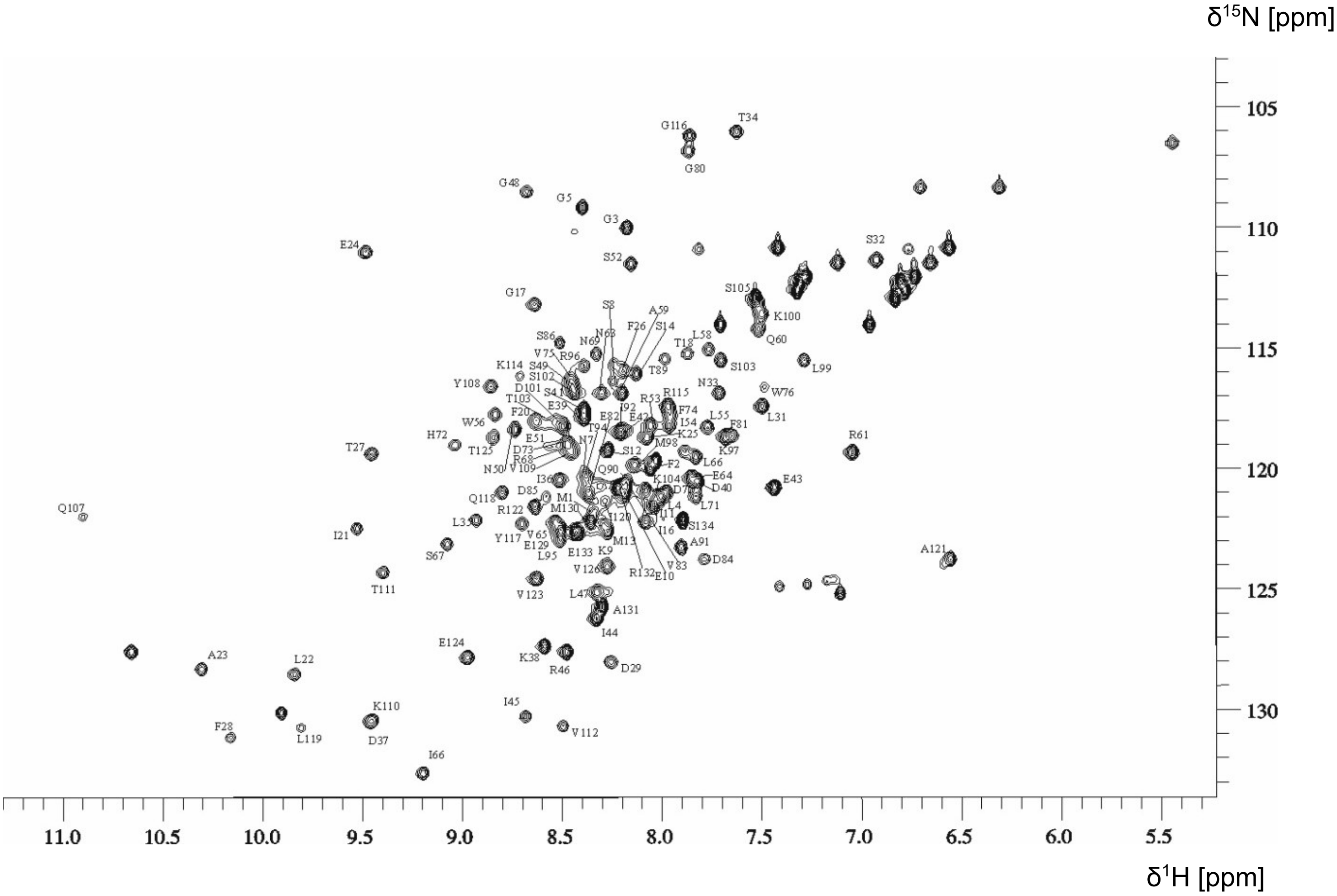

Supplementary Figure 2 Amino acid sequence of cToxR_1-134. Underlined residues could not be assigned in the 2D ^{15}N-^{1}H HSQC.

Supplementary Table 2 List of manually assigned NOEs of cToxR_1-134 characterized by their distance in the amino acid sequence.

Residues	NOE type
Ile 92 Hd1* - Tyr 117 He*	Very Long range *(i - i+25-100)*
Tyr 117 H – Ile 66 H	Very long range
Ala 121 Hb – Ala 23 H	Very long range
Val 126 H – Phe 20 Ha	Very long range
Val 123 Ha – Leu 22 Ha	Very long range
Gln 124 H – Ile 21 H	Very long range
Leu 88 Hda – Ile 92 Hd1*	Long range *(i – i+4-9)*
Leu 88 Hg - Ile 92 Hd1*	Long range
Tyr 108 Hd* - Leu 99 Hda*	Long range
Gly 116 H – Val 112 H	Long range
Val 112 H – Tyr 117 Ha	Long range
Val 112 Hga* - Tyr 117 Ha	Long range
Thr 111 Ha – Tyr 117 Ha	Long range
Ile 66 Hd1* - Arg 61 H	Long range
Ile 92 Hd1* - Thr 89 Ha	Short range *(i – i+2-3)*
Val 65 Ha – Ile 66H	Short range
Ala 121 H – Leu 119 Ha	Short range
Gln 107 H – Tyr 108 H	Sequential *(i – i+1)*
Tyr 108 H – Val 109 H	Sequential
Ile 21 H – Phe 20 Ha	Sequential
Gln 24 H – Ala 23 H	Sequential
Pro 106 Hb – Gln 107 H	Sequential
Leu 32 Ha – Ala 23 H	Sequential
Gln 107 H – Val 109 H	Sequential
Gln 118 H – Tyr 117 Ha	Sequential
Leu 22 Ha – Ala 23 Ha	Sequential
Ala 121 H – Ile 120 H	Sequential
Ala 121 H – Arg 122 H	Sequential
Ala 121 H – Ile 120 Ha	Sequential
Ala 121 H – Ile120 Hg1b	Sequential

DATA SEQUENCE	MFGLGHNSKE	ISMSHIGT**KF**	**IL**AEK**FTFD**P	LSN**TLI**DKED	SEE**IIRL**GSN
DATA PREDICTED	LLLLLLLLLL	LLLLLLLL**EE**	**EE**LLL**EEEE**L	LLL**EEE**LLLL	LLL**EEEE**LHH
DATA CONFIDENCE	8788999987	89987698**08**	**98**1643**880**6	476**697**2788	871**6773**869
DATA SEQUENCE	ESRILWLLAQ	RPNE**VIS**RND	LHDFVWREQG	FEVDDSSLTQ	AISTLRKMLK
DATA PREDICTED	HHHHHHHHHH	LLLL**EEE**HHH	HHHHHHXXXL	LLLLHHHHHH	HHHHHHHHLL
DATA CONFIDENCE	9999999982	7897**441**699	9999990008	6568389999	9999999718
DATA SEQUENCE	DSTKSPQ**YVK**	**TV**PKR**GYQLI**	A**RVETV**EEEM	ARES	
DATA PREDICTED_SS	LLLLLLL**EEE**	**EE**LLL**EEEEE**	L**EEEEE**XXLL	LLLL	
DATA CONFIDENCE	8826981**789**	**70**844**79997**	2**38973**0089	8880	

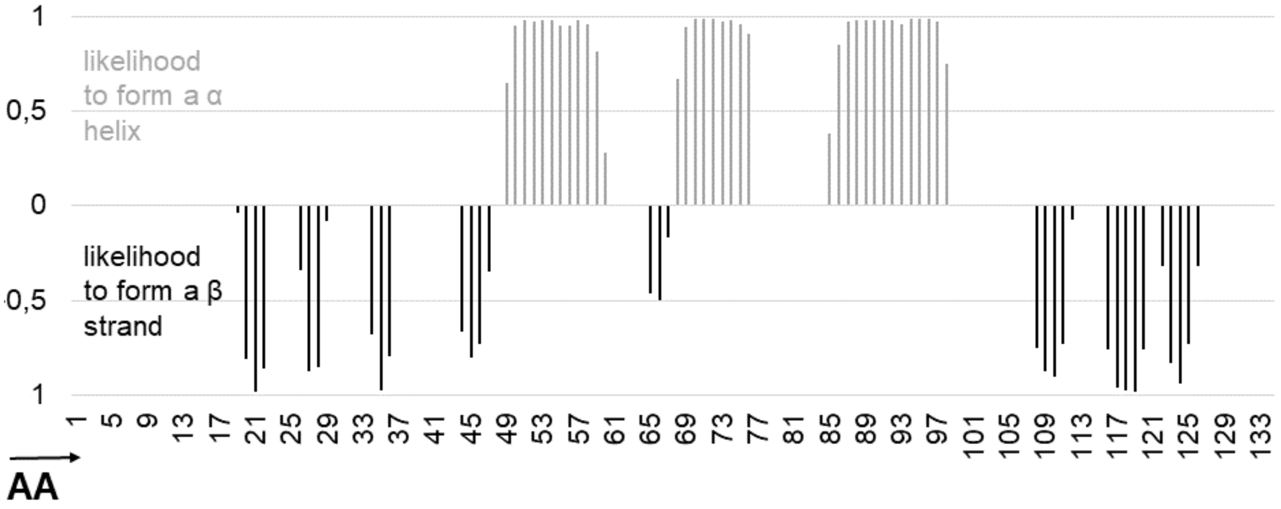

Supplementary Figure 3 Talos+ (Shen et al. 2009a) secondary structure prediction of cToxR_1-134 using NMR chemical shift information. The likelihood for each residue to form the predicted secondary structure (E = strand, H = helix, L = loop) is shown underneath each residue. The residues involved in helices are highlighted in light grey, residues forming sheets are written in bold. The graphical representation of the prediction is shown below, residues involved in helix formation are shown in light grey, β-sheet forming residues are shown in black.

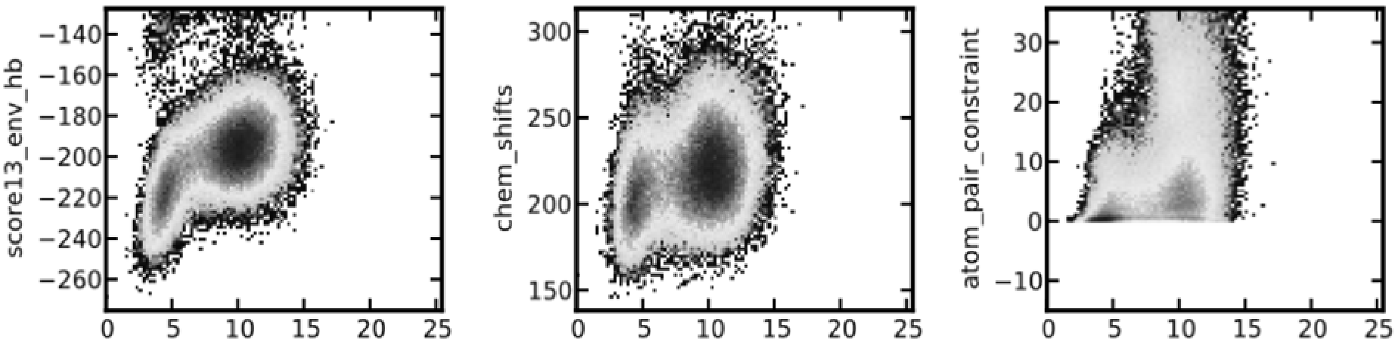

Supplementary Figure 4 Scores of the 100.000 structure models obtained from CS-Rosetta are shown. From left to right, the Rosetta score score13_env_hb, the chemical shift score and the atom pair constraint are plotted against the RMSD (Å) to the best-scored structure model. All three scores correlate well with the RMSD, indicating that the algorithm has converged.

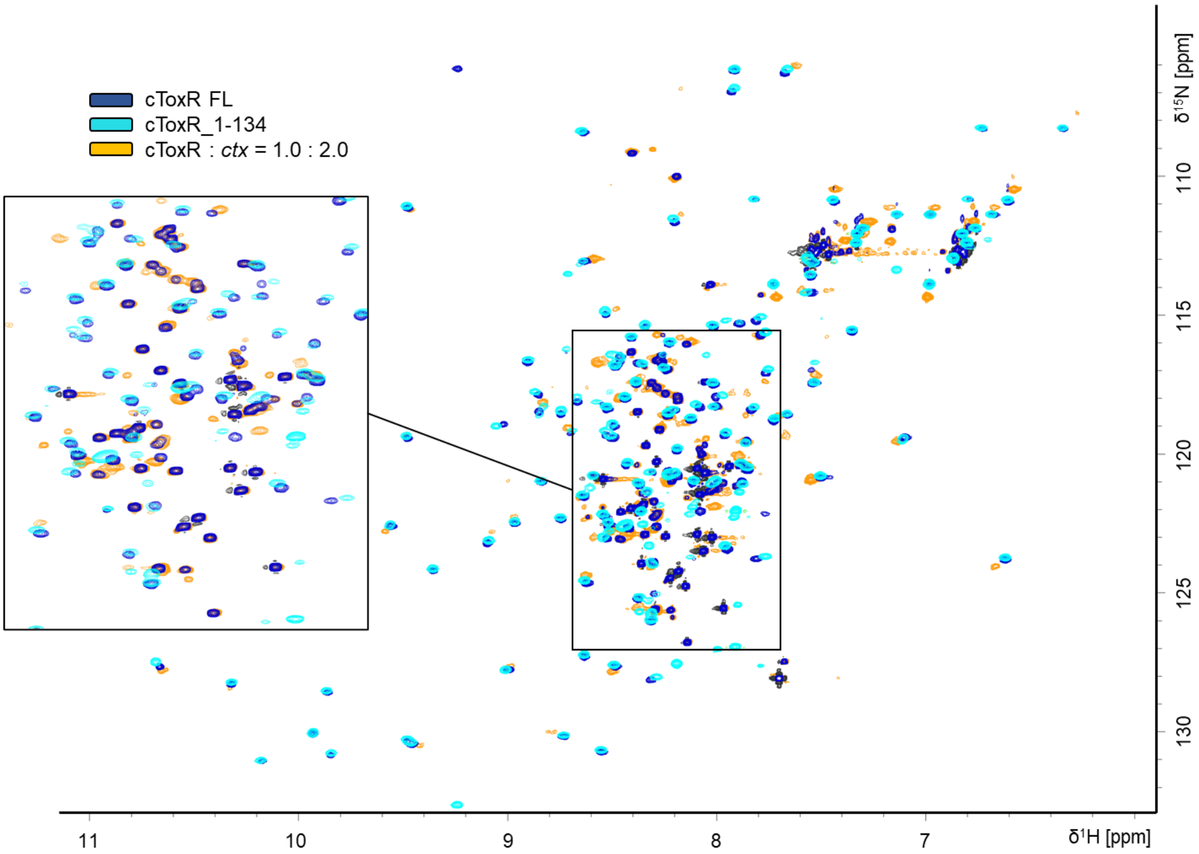

Supplementary Figure 5 ^{15}N-HSQC NMR experiments with full-length FL cToxR before (dark blue) and after the addition of *ctx* DNA (orange). ^{15}N-HSQC of the C-terminal truncated construct cToxR_1-134 is shown in skyblue. Peaks that appear only in the cToxR FL spectrum (dark blue) not in the truncated construct cToxR_1-134 (sykblue) are located in the middle unstructured region of the spectrum (zoom) and reveal no change of the chemical shift upon addition of DNA (orange), suggesting no involvement in the interaction with DNA.

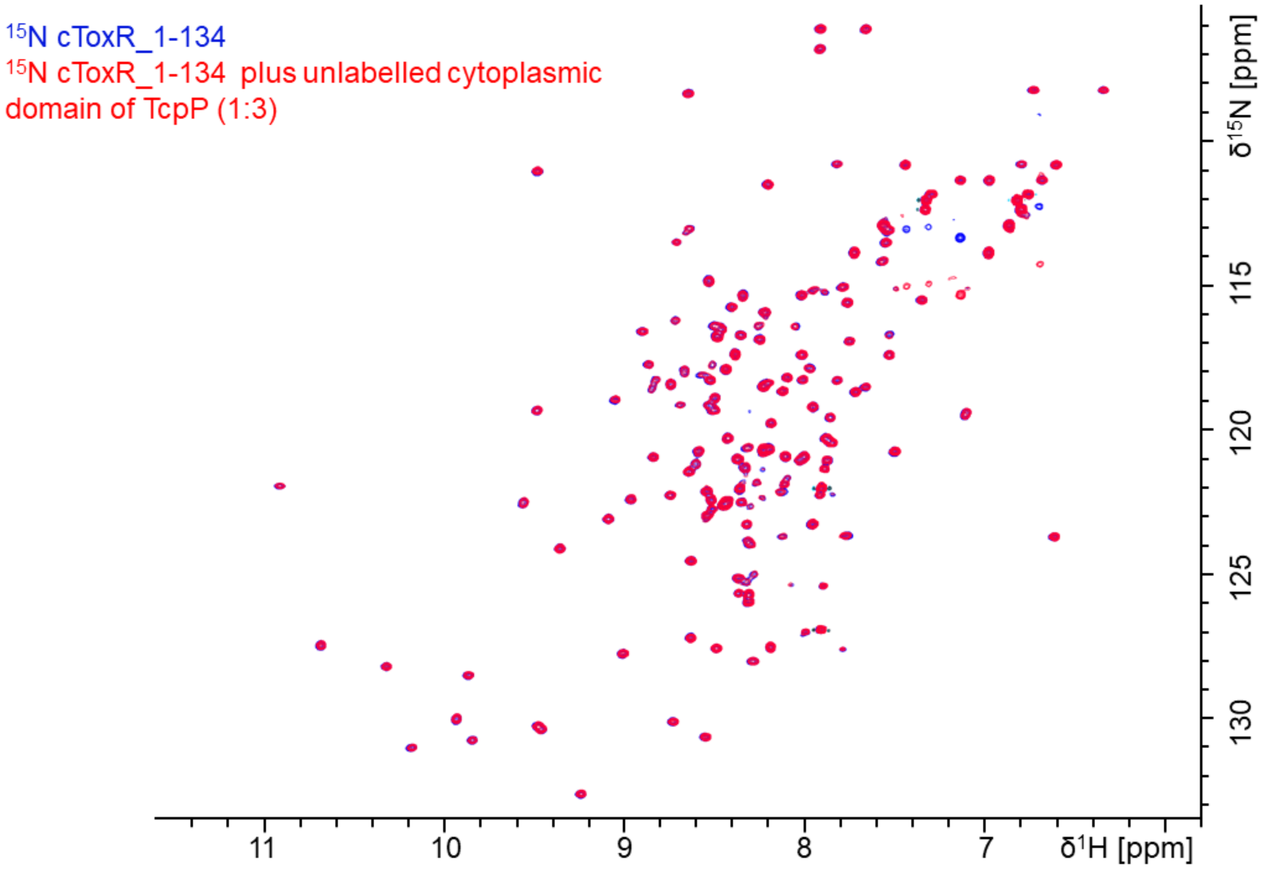

Supplementary Figure 6 ^{15}N-HSQC experiment with the cytoplasmic domains of ToxR (blue) and TcpP (red). TcpP was added in a three-molar excess over cToxR. We could not observe significant chemical shift changes, therefore concluding no binding event is happening under the experimental conditions.

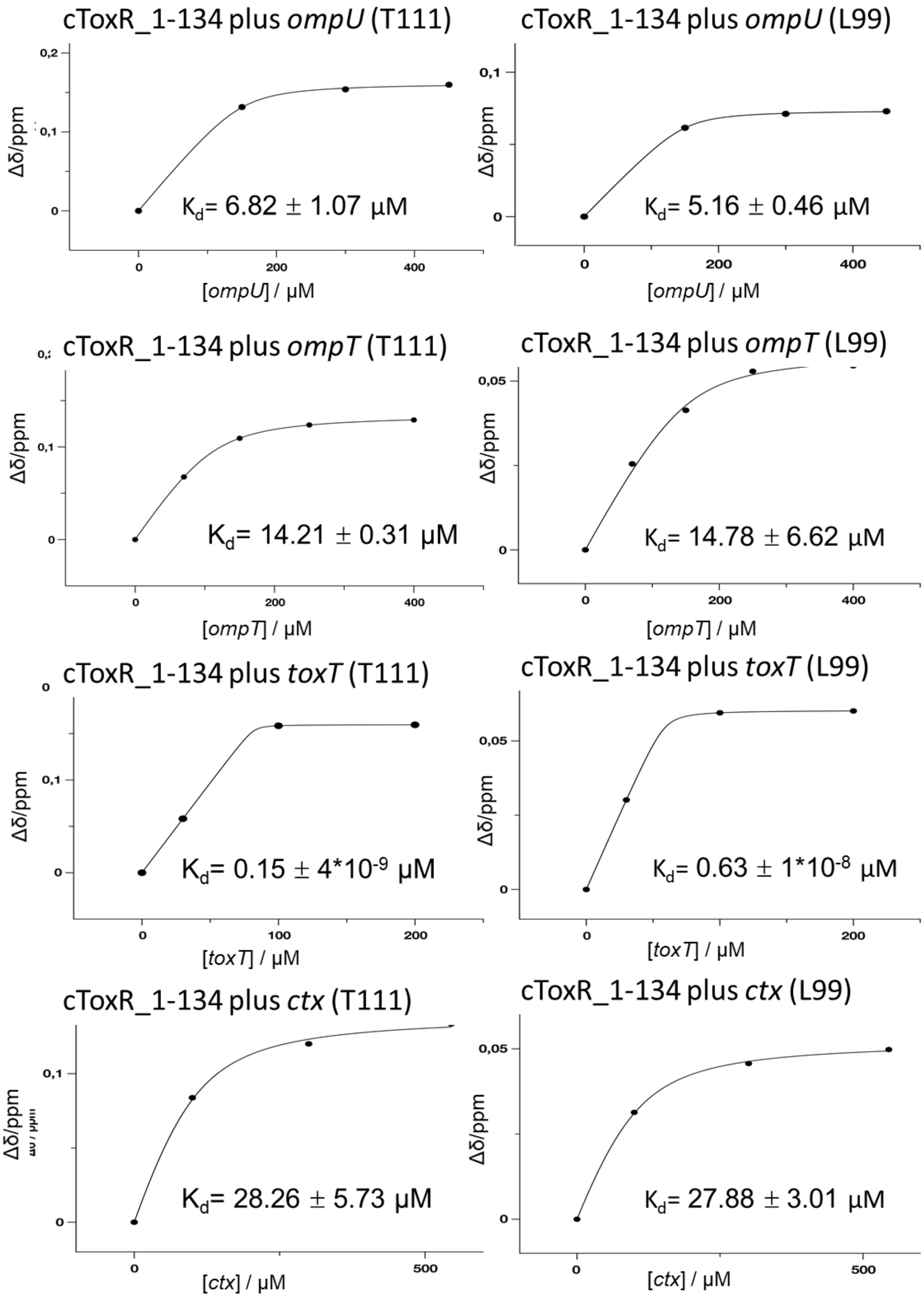

Supplementary Figure 7 Determination of NMR based dissociation constants between cToxR_1-134 and double-stranded DNA motifs: *ompU, ompT*, *toxT* and *ctx*.The calculation was done using changes of chemical shift values of T111 and L99 upon addition of DNA. Experimental setups are described in the 'Materials and methods' section.

cToxR_1-134 plus *ompU*

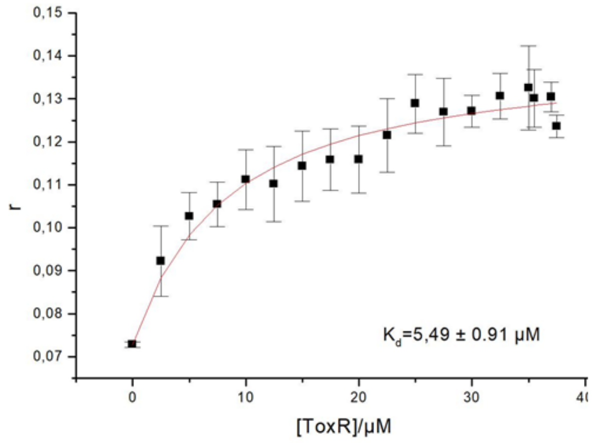

cToxR_1-134 plus *ompT*

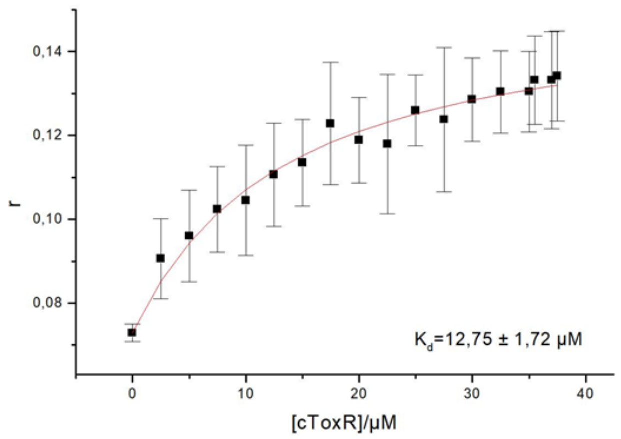

cToxR_1-134 plus *toxT*

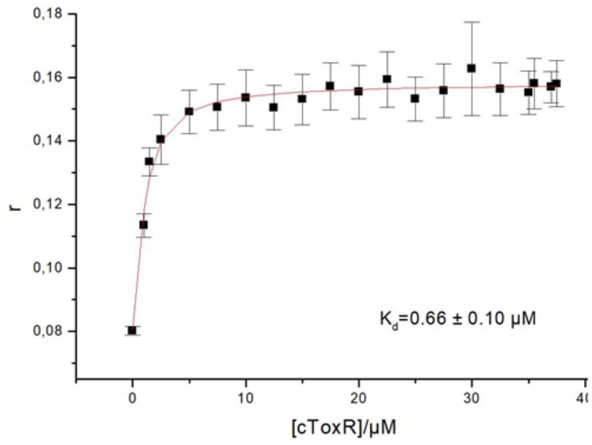

cToxR_1-134 plus *ctx*

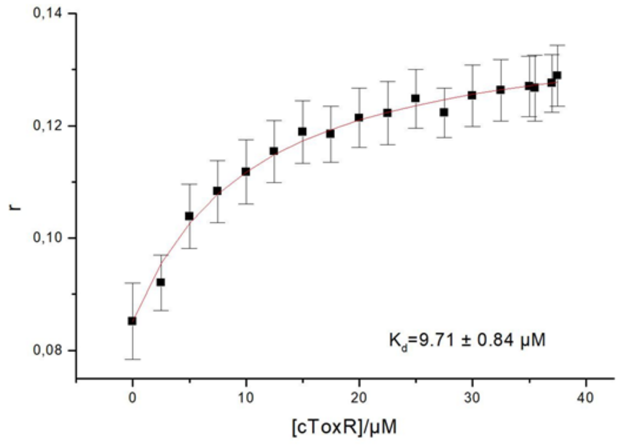

cToxR_1-134 plus DNA

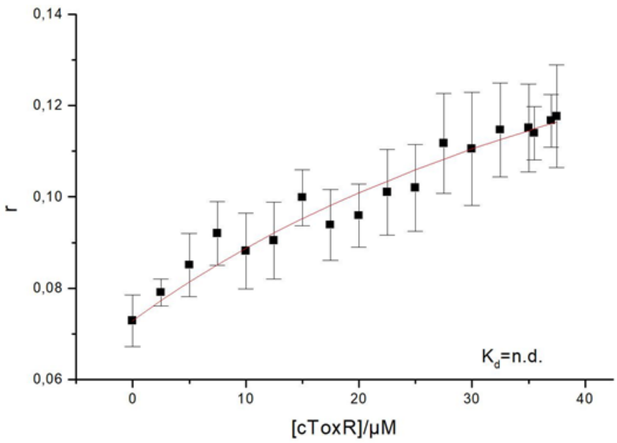

Supplementary Figure 8 Determination of dissociation constants between cToxR_1-134 and double-strandedDNA motifs by fluorescence anisotropy: *ompU, ompT, toxT* and *ctx*, as well as a randomly selected DNA strand. Base sequences are listed in Table 1, experimental procedures are described in the ‘Materials and Methods’ section.

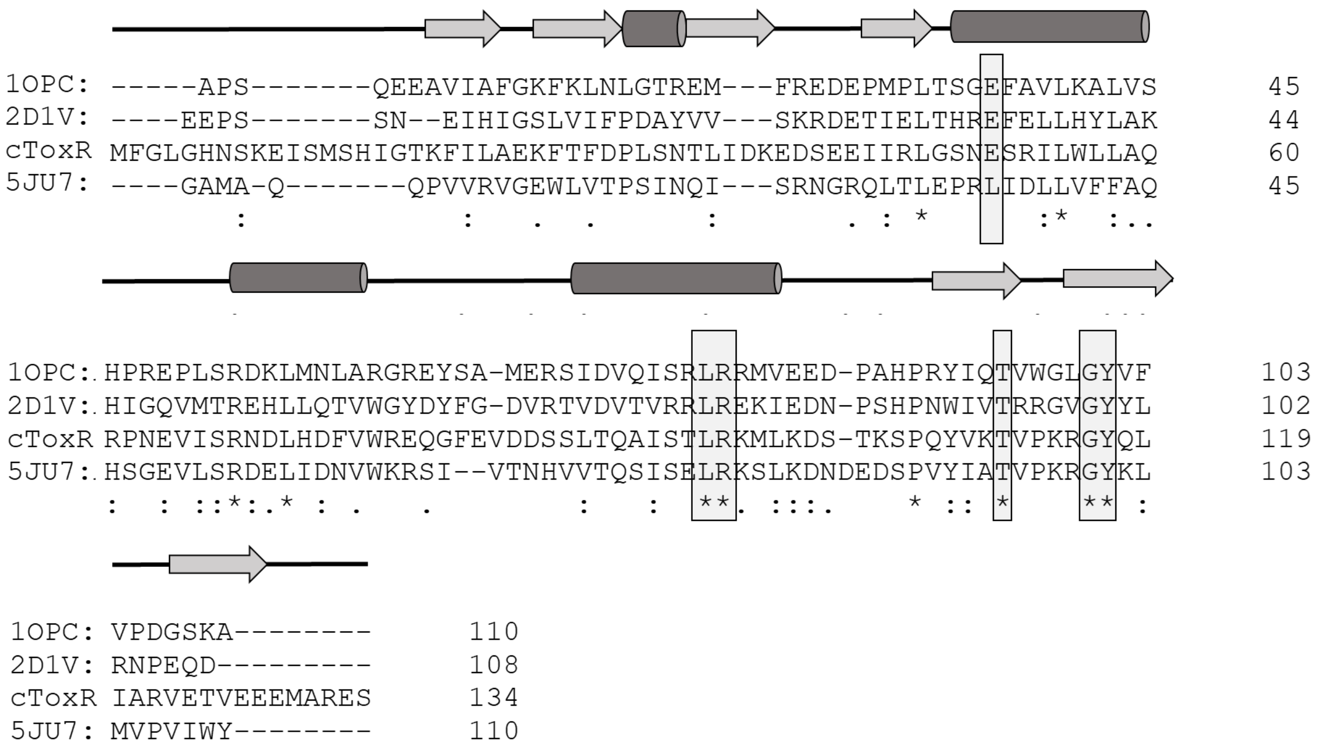

Supplementary Figure 9 Multiple sequence alignment (Clustal Omega (Madeira et al. 2019)) between wHTH proteins cToxR, cYycF (*Bacillus subtilis*, PDB code: 2d1v), cOmpR (*E.coli*, pdb code: 1opc) and CadC (*E.coli*, pdb code: 5ju7). Conserved residues are highlighted, the secondary structure elements of cToxR_1-134 are displayed above the sequence. Amino acids that are essential for the formation of the protein DNA complex are conserved and mostly located nearby the HTH motif and wing 1.

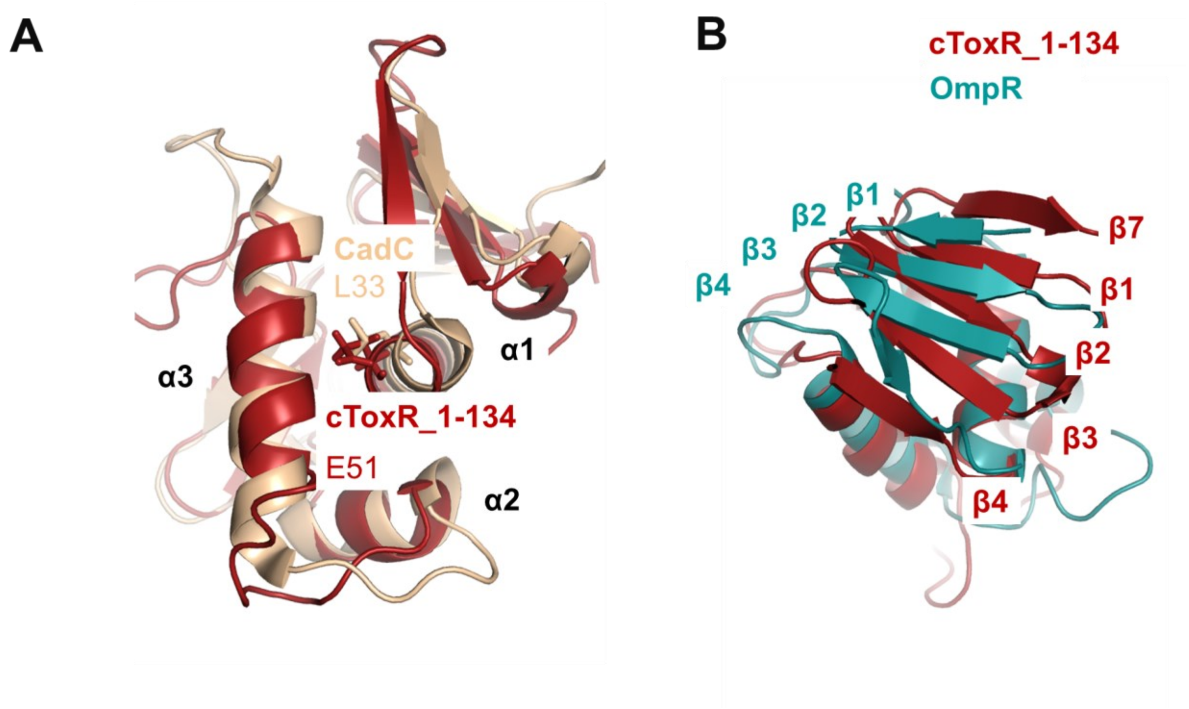

Supplementary Figure 10 A Structural overlay of 'ToxR-like' proteins cToxR_1-134 from *V. cholerae* (red) and CadC from *E. coli* (beige). The conserved E51 residue (shown in sticks) is part of helix 1 and stabilizes the positioning of the recognition helix 3. CadC contains a leucine residue instead which is positioned in a similar way. B: Structural overlay of wHTH proteins cToxR_1-134 (red) and OmpR from *E. coli* (turquois). In contrast to OmpR, cToxR_1-134 forms a five stranded β sheet consisting of four N-terminal β strands (β1-β4) and β-strand 7. OmpR contains only a four stranded N-terminal β sheet (β1-β4).
